# Video-Oculography During Free Visual Exploration to Detect Right Spatial Neglect in Left-Hemispheric Stroke Patients With Aphasia: A Feasibility Study

**DOI:** 10.3389/fnins.2021.640049

**Published:** 2021-03-29

**Authors:** Brigitte C. Kaufmann, Dario Cazzoli, Monica Koenig-Bruhin, René M. Müri, Tobias Nef, Thomas Nyffeler

**Affiliations:** ^1^Perception and Eye Movement Laboratory, Department of Neurology, University of Bern, Bern, Switzerland; ^2^Neurocenter, Luzerner Kantonsspital, Lucerne, Switzerland; ^3^CNRS, Institut du Cerveau–Paris Brain Institute–ICM, Inserm, Sorbonne Université, Paris, France; ^4^Gerontechnology and Rehabilitation Group, ARTORG Center for Biomedical Engineering Research University of Bern, Bern, Switzerland

**Keywords:** left-hemispheric stroke, right spatial visual neglect, aphasia, video-oculography, free visual exploration, mean gaze position, visual exploration behaviour

## Abstract

Spatial neglect has been shown to occur in 17–65% of patients after acute left-hemispheric stroke. One reason for this varying incidence values might be that left-hemispheric stroke is often accompanied by aphasia, which raises difficulties in assessing attention deficits with conventional neuropsychological tests entailing verbal instructions. Video-oculography during free visual exploration (FVE) requires only little understanding of simple non-verbal instruction and has been shown to be a sensitive and reliable tool to detect spatial neglect in patients with right-hemispheric stroke. In the present study, we aimed to investigate the feasibility of FVE to detect neglect in 10 left-hemispheric stroke patients with mild to severe aphasia as assessed by means of the Token Test, Boston Naming Test and Aachener Aphasie Test. The patient’s individual deviation between eye movement calibration and validation was recorded and compared to 20 age-matched healthy controls. Furthermore, typical FVE parameters such as the landing point of the first fixation, the mean gaze position (in ° of visual angle), the number and duration of visual fixations and the mean visual exploration area were compared between groups. In addition, to evaluate for neglect, the Bells cancellation test was performed and neglect severity in daily living was measured by means of the Catherine Bergego Scale (CBS). Our results showed that the deviation between calibration and validation did not differ between aphasia patients and healthy controls highlighting its feasibility. Furthermore, FVE revealed the typical neglect pattern with a significant leftward shift in visual exploration bahaviour, which highly correlated with neglect severity as assessed with CBS. The present study provides evidence that FVE has the potential to be used as a neglect screening tool in left-hemispheric stroke patients with aphasia in which compliance with verbal test instructions may be compromised by language deficits.

## Introduction

Spatial neglect, as assessed with classic neuropsychological paper-pencil tests, has been reported to occur in 17–65% of patients after left-hemispheric stroke (Stone et al., 1993; Beis et al., 2004; Ringman et al., 2004; Beume et al., 2017). One important reason for this varying incidence values might be that testing for neglect in left-hemispheric stroke patients is often hindered by aphasia. Indeed, impaired understanding of verbal instructions may already limit the administration of relatively simple neuropsychological paper-pencil tests, such as cancellation and line bisections tests (Bowen et al., 1999), and even more considerably for more complex testing procedures. In addition, language deficits are often an exclusion criterion in studies investigating neglect after left-hemispheric stroke (e.g., Stapleton et al., 2000; Chung et al., 2016), leading to a very limited knowledge about visuospatial attentional deficits in this particularly vulnerable patient group.

In patients with right-hemispheric stroke, video-oculography during free visual exploration (FVE) has increasingly become a valuable screening tool to detect left-sided neglect after a right-hemispheric lesion. In this context, the assessment of the spatial distribution of visual fixations along the horizontal axis during FVE, reflecting the spatial allocation of visual attention, has proved as a particularly suitable indicator (e.g., Pflugshaupt et al., 2004; Nyffeler et al., 2008; Ptak et al., 2009; Cazzoli et al., 2011; Osandón et al., 2012; Müri et al., 2013; Fellrath and Ptak, 2015; Paladini et al., 2019; Kaufmann et al., 2020a, b). Visual exploration is a spontaneous behaviour; hence, the calibration of an eye-tracking system and its validation, as well as the measurement of eye movements during an FVE task, require only few and simple verbal task instructions. It seems thus reasonable to assume that eye-tracking during FVE can be successfully applied in patients with aphasia after a left-hemispheric lesion in order to detect right-sided neglect.

The aim of the present study was to test this hypothesis, i.e., administer a commonly used FVE paradigm (Delazer et al., 2018; Paladini et al., 2019; Kaufmann et al., 2020a) in order to assess the feasibility of applying video-oculography to detect right-sided neglect in left-hemispheric stroke patients with aphasia.

## Materials and Methods

### Patients and Healthy Controls

**FIGURE 1:**
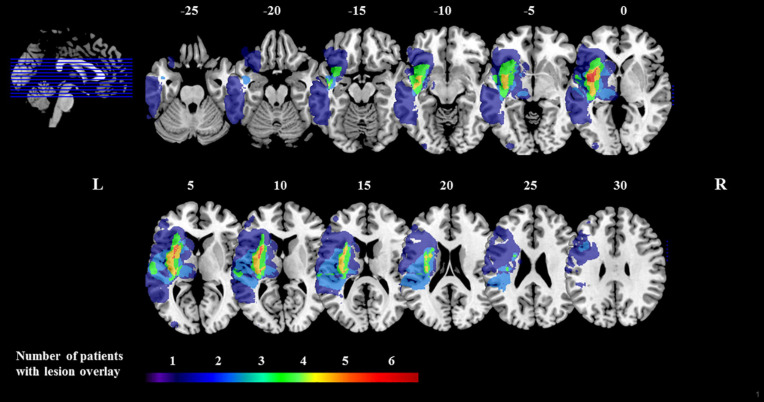
Lesion overlay plot. Brain lesions of all 10 patients with left-hemisphere stroke. The color-coded legend is determined by the number of patients with damage to a specific brain region. Lesion overlap maps are plotted on the CH2bet template available in MRIcron (https://www.nitrc.org/projects/mricron). Axial slices are oriented according to the neurological convention. The z-position of each axial slice, in MNI coordinates, is indicated by the numbers at the top of the respective slices, and also depicted by the blue lines on the sagittal slice (top, left-hand side of the figure).

The anonymised data of 10 patients with aphasia after left-hemispheric sub-acute stroke was included in this study (4 females; mean age = 66.70, *SD* = 13.03; mean time since stroke = 34.3 days, *SD* = 27.66). A lesion overlay plot is shown in Figure 1, individual demographic and clinical patient data are shown in Table 1. All patients showed mild to severe aphasia at the time-point of measurement.

**TABLE 1:** Table shows patients’ individual demographic data (sex, handedness, agerange, time since stroke in days), their individual right-sided neglect severity score in the activities of daily living, as assessed by the Catherine Bergego Scale (CBS), as well as their individual scores in the neuropsychological paper-pencil test (Bells Test), the mean gaze position on the horizontal axis in Video-Oculography, and their individual scores in the linguistic assessments of aphasia [Token Test, Boston Naming Test, and scores for the subscale “Sprachverständnis” (i.e., speech comprehension) of the Aachener Aphasia Test (AAT) based on the Standard Nine Scale (Huber et al., 1983)].

**Patient**	**Sex**	**Handedness**	**Age (range)**	**Time since stroke (days)**	**Token test score**	**Boston naming test score**	**Aachener aphasie test (severity of comprehension disorder)**	**CBS score at admission**	**Bells test (CoC)**	**Mean gaze position (in °)**
Patient 1	w	R	51–60	26	**7**	**8**	Not feasible	**3**	0.019	–1.305
Patient 2	w	R	51–60	33	**0**	**0**	**Moderate**	**5**	–0.033	**−2.145**
Patient 3	m	R	71–80	22	8	**5**	**Mild**	**2**	0.000	–0.170
Patient 4	m	L	51–60	108	**6**	**13**	**Mild**	0	–0.010	0.211
Patient 5	m	R	81–90	48	**5**	**0**	**Severe**	**5**	0.002	**−3.044**
Patient 6	w	R	81–90	28	8	14	**Mild**	**7**	**−0.133**	**−2.467**
Patient 7	m	R	61–70	25	**0**	**0**	**Moderate**	**10**	0.053	**−2.493**
Patient 8	w	R	81–90	25	**0**	**0**	**Moderate**	0	–0.075	–0.753
Patient 9	m	R	51–60	12	8	**10**	**Moderate**	0	0.000	0.052
Patient 10	m	R	51–60	16	**0**	**0**	**Moderate**	0	0.041	0.709
Mean (SD)			66.70	34.3	4.2	5		3.20	–0.019	–1.141
			(13.03)	(27.66)	(3.74)	(5.81)		(3.49)	(0.064)	(1.334)

**Clinically significant test scores indicating the presence of Aphasia and Neglect are indicated in bold.**

All patients signed a written informed general consent allowing the retrospective use of their anonymised health related data for research purposes. Additional ethical review and approval was not required for the study on human participants in accordance with the local legislation and institutional requirements. Additionally, video-oculography data of 20 healthy, age-matched control participants (described below in the paragraph Data Pre-processing and Statistical Analyses), was used to investigate the feasibility of video-oculography. The data of healthy control participants has been previously included in a methodological paper, which was reviewed and approved by the Ethics Committee Nordwest and Zentralschweiz (EKNZ), Switzerland. Written informed consent to participate in this study was provided by all healthy participants.

### Linguistic and Neuropsychological Assessments

For the linguistic assessments in left-hemispheric stroke patients, we identified patients as having aphasia if they presented with pathological performance in the Token Test (Lang et al., 1999) and/or the short version of the Boston Naming Test (Morris et al., 1988) and/or the Aachener Aphasia Test (Huber et al., 1983).

Aphasia was defined according to current, established cut-off scores: for the Token Test (number of correct stimuli), 0–4 severe aphasia, 5–7 mild aphasia, 8–10 no aphasia (Lang et al., 1999); for the Boston Naming Test (short version including 15 items, number of correct stimuli), cut-off values were corrected for age and education according to Morris et al. (1988); for the different subtests of the Aachener Aphasia Test, normative values were based on Huber et al., 1983).

A systematic, ecological observation questionnaire during everyday behaviour, i.e., the Catherine Bergego Scale (CBS), was used to diagnose neglect (Azouvi et al., 2003). Neglect in daily living was defined according to current, established cut-offs scores (CBS score 1–10 mild neglect, 11–20 moderate neglect, 21–30 severe neglect). Furthermore, Cancellation tasks [e.g., the Bells Cancellation Test (Gauthier et al., 1989)] are also commonly applied in order to identify spatial neglect in both clinical and experimental settings (Azouvi et al., 2002; Beis et al., 2004; Rorden and Karnath, 2010). Cancellation tasks have also been shown to be a valuable tool to diagnose neglect in left-hemispheric stroke patients (Suchan et al., 2012). Therefore, in the present study, neglect was additionally characterised according to current, established cut-off scores for the Bells Cancellation test, i.e., centre of cancellation (CoC) <−0.086 (Suchan et al., 2012).

### Practical Aspects of Video-Oculography

#### Video-Oculography Set-Up

**FIGURE 2:**
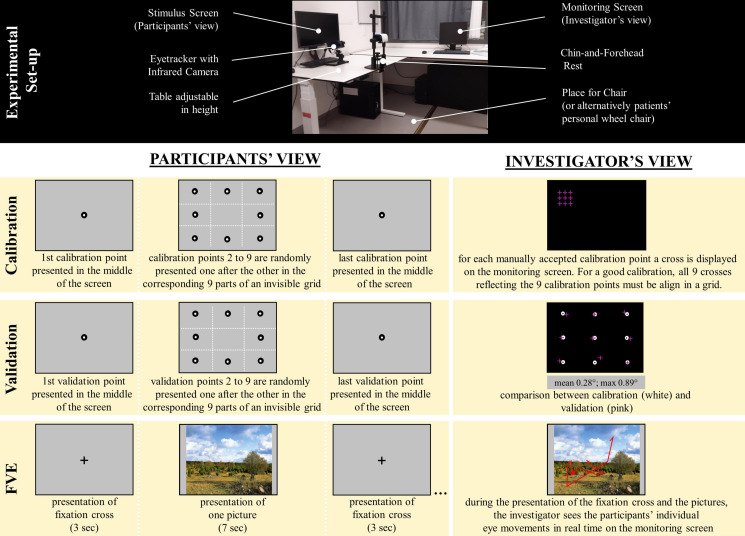
Experimental set-up of FVE and Participants’/Investigators’ View during FVE. **Top** Experimental Set-up of the video-oculography equipment, consisting of a stimulus screen, on which the FVE paradigm is presented, and a Monitoring Screen, on which the investigator can monitor the patients’ individual eye movements during testing. Eye movements are recorded using an infrared-based camera (e.g., Eyelink 1000 Plus by SR research). A height-adjustable table can be adapted to find the optimal configuration of the experimental set-up for each patient. Furthermore, a height-adjustable table allows examining patients in a wheel-chair, wherefore usually additional table height is needed to fit the armrests. A chin-and-forehead-rest was used to both ensure that the mid-sagittal plane of the patients was aligned with the middle of the screen at a constant distance, and to minimise head movements. **Bottom Left** shows the participants’ view of the stimulus screen during Calibration (top), Validation (middle), and during the Free Visual exploration (FVE) Paradigm (bottom). **Bottom Right** shows the Investigator’s View on the monitoring screen during Calibration, Validation, and during FVE.

The eye-tracking system consists of three parts: (1) a remote, infrared-based, video eye-tracking system (EyeLink 1000 Plus System, SR Research, Ottawa, Canada); (2) a stimulus screen, in front of the participant, on which all the stimuli were presented; and, (3) a monitoring screen for the investigator, on which the participants’ eye movements are shown in real-time (Figure 2, top).

During video-oculography, caution needs to be exercised in the correct positioning of the patients, in order to obtain qualitatively high-standard data. Hereby, a chin-forehead-rest was used to both ensure that the patients’ mid-sagittal plane was aligned with the middle of the stimulus screen at a constant distance and to minimise head movements. A height-adjustable table was used to optimally adapt the height of the experimental set-up for each patient. Furthermore, a height-adjustable table allows to examine patients in a wheel-chair, wherefore usually additional table height is needed to fit the armrests of the latter.

Due to the variably impaired verbal language comprehension abilities of aphasia patients, we adapted the task instructions to the patients’ individual needs. We therefore used very simple sentences and/or gestures to explain the investigation on a step-by-step basis. Furthermore, it is often of advantage to demonstrate the procedure in a non-verbal way, i.e., the examiner concretely performs the actions that the patient is then asked to perform him/herself. In the following, we present how the examination can be explained to patients with language impairments.

#### Video-Oculography Instruction and Paradigm

After welcoming the patient, the investigator first communicates him/her that eye-movements are going to be measured, also pointing to the eye-tracking camera and the eyes. Then, the investigator shows how the FVE will be performed. First, the investigator takes a seat in front of the eye-tracking camera and positions the head on the chin-forehead-rest, the arms lying comfortably on the table, as expected to be done by the patient later on. Then, the investigator shows how the height-adjustable table is operated in order to find the optimal height of the experimental set-up. After describing verbally the process in simple sentences, as well as providing a concrete example of the seating position, the patient is invited to copy the position showed by the investigator. By means of this procedure, all patients in this study were able to understand how to position themselves.

After an optimal seating position was reached, video-oculography was started and the patients were guided through the 5 min task, during which eye movements were recorded.

In order to correctly measure eye movements, the eye-tracking system needs to be individually calibrated; moreover, in order to guarantee data quality, this calibration needs to be validated. The calibration procedure started by a black dot with a white centre, presented in the middle of the stimulus screen on a light grey background. If the patient steadily looked at the dot, the investigator manually confirmed this position to the system. Afterward, 9 further dots were presented, one after the other, at different positions corresponding to an invisible 9-point grid (3 × 3; see Figure 2, bottom). If, at any time, a patient did not automatically fixate the suddenly appearing points (i.e., the patients gaze was wandering around), his/her attention was drawn to the corresponding point by simply pointing at it with the finger. The advantage of this procedure is that the suddenly appearing black dots, individually presented on a grey background, tend to spontaneously draw the gaze, usually not needing explicit instructions. Indeed, eight out of the 10 patients performed the calibration procedure without external instructions. After calibration, the exactly same procedure was repeated for the purpose of validation; thereby, the system computed the mean deviation between gaze position during calibration and validation. If the mean deviation between calibration and validation was >1°, then the computer suggested to repeat the procedure to improve the values.

The mean deviation values between calibration and validation indicating high-standard measurement quality (i.e., ≤1°) were achieved in all patients. These individual values were included in the statistical analyses as quality indicators.

After calibration and validation, the FVE paradigm was introduced. Six pictures of natural scenes or urban public places, and their 6 mirrored versions (mirrored along the central, vertical axis), were presented on the stimulus screen (Paladini et al., 2019; Kaufmann et al., 2020a, b). The pictures were presented for 7 s each. Between the pictures, a central, black fixation-cross on a grey background was displayed for 3 s, in order to enforce a common central starting point of visual exploration for all participants. Because visual exploration occurs spontaneously, this task did not need any particular instruction (Figure 2, bottom).

### Data Pre-processing and Statistical Analyses

#### Feasibility Characteristics

To evaluate the feasibility of video-oculography, the patients’ individual deviation between calibration and validation was recorded and compared to the one of 20 healthy, age-matched control participants [9 women; no significant age difference between groups: Patients mean = 66.70 (SD = 13.03), Healthy mean = 66.60 (SD = 10.10); *t*(28) = 0.0230, *p* > 0.05; healthy controls have been previously included in a methodological paper (Kaufmann et al., 2020b)] using a *t*-test for independent groups. Furthermore, the spontaneous feedback of the patients and, if available, of their relatives was documented.

#### Video-Oculography Data

Pearson’s correlations were computed (*p*-values 2-tailed) between the mean gaze position and neglect severity in everyday behaviour, as assessed by means of the CBS, in left-hemispheric stroke patients. Additionally, Pearson’s correlations was computed between the two neglect measures (i.e., the CBS and the mean gaze position) and the two linguistic assessments, i.e., the test performance in the Token Test (Lang et al., 1999) and the short version of the Boston Naming Test (Morris et al., 1988). To control for multiple testing in the five correlations, significant levels were Bonferroni-corrected resulting in a threshold of *p* = 0.01.

For FVE, the presence of neglect was defined according to the spatial distribution of fixations, i.e., a mean gaze position (in ° of visual angle) on the horizontal axis of <−1.359°, indicating a leftward-shifted spatial distribution of fixations (Kaufmann et al., 2020a). The mean gaze position was compared between the 10 patients with aphasia after left-hemispheric stroke and the 20 healthy, age-matched control participants.

Previous studies (e.g., Pflugshaupt et al., 2004; Kaufmann et al., 2020a) found that early attentional orienting is good predictor of neglect. Hence, in the present study, the landing point of the first fixation on each picture (left or right screen half) was determined, and the percentage of left-ward first fixations was computed for each participants and compared between groups using an independent *t*-test.

Additional response variables concerning video-oculography (i.e., number of fixations per screen half, fixation time spent within each screen half, and visual exploration area within each screen half) were compared between the two groups and the screen halves by means of a 2 × 2 mixed-model ANOVA with the between factor group (2 levels: healthy controls, neglect patients) and the within factor screen half (2 levels: left and right). Bonferroni-corrected *post hoc t*-tests were used to compare the results between the different combinations of factor levels.

The significance level for all statistical tests was set to alpha = 0.05. All fixations with duration between 100 and 2,000 ms were included in the off-line data analyses (Salthouse and Ellis, 1980; Carpenter, 1988; Kaufmann et al., 2020a, b), which resulted in the exclusion of 4.89% of all fixations.

## Results

### Linguistic and Neuropsychological Assessments

The linguistic assessments (Token Test, Boston Naming Test, and Aachener Aphasie Test), showed mild to severe aphasia in all 10 patients (Token Test mean = 4.20, SD = 3.74, range 0–8 out of 10; Boston Naming Test mean = 5.00, SD = 5.81, range 0–14 out of 15). In 6 out of 10 patients, the Catherine Bergego Scale revealed mild right-sided neglect in daily living (CBS mean = 3.20, *SD* = 3.49; range 0–10). The Bells cancellation test revealed right-sided neglect in 1 out of 10 patients (mean CoC = −0.019, *SD* = 0.064). Individual test scores for each patient are shown in Table 1.

### Feasibility Characteristics

In all patients, the mean deviation between calibration and validation was within the range of good quality, as indicated by the device manufacturer (Mean = 0.448, *SD* = 0.232; no significant difference between patients with mild aphasia (mean = 0.503, *SD* = 0.332) and patients with moderate/sever aphasia [mean = 0.412, *SD* = 0.163; unpaired *t*(8) = 0.586, *p* = 0.574]. None of the measures had to be repeated. Furthermore, the spontaneous feedback of the patients and their relatives was very positive. While the patients seemed relaxed and calm, the patients’ relatives reported that they had the feeling that the patients had felt comfortable during the measurements.

A *t*-test for independent groups, comparing the deviation between calibration and validation between healthy participants and left-hemispheric stroke patients, revealed no significant difference between groups (mean in healthy control participants = 0.449, *SD* = 0.194, mean in patients = 0.473 (*SD* = 0.251); *t*(28) = −0.296, *p* = 0.770).

### Video-Oculography Data

**FIGURE 3:**
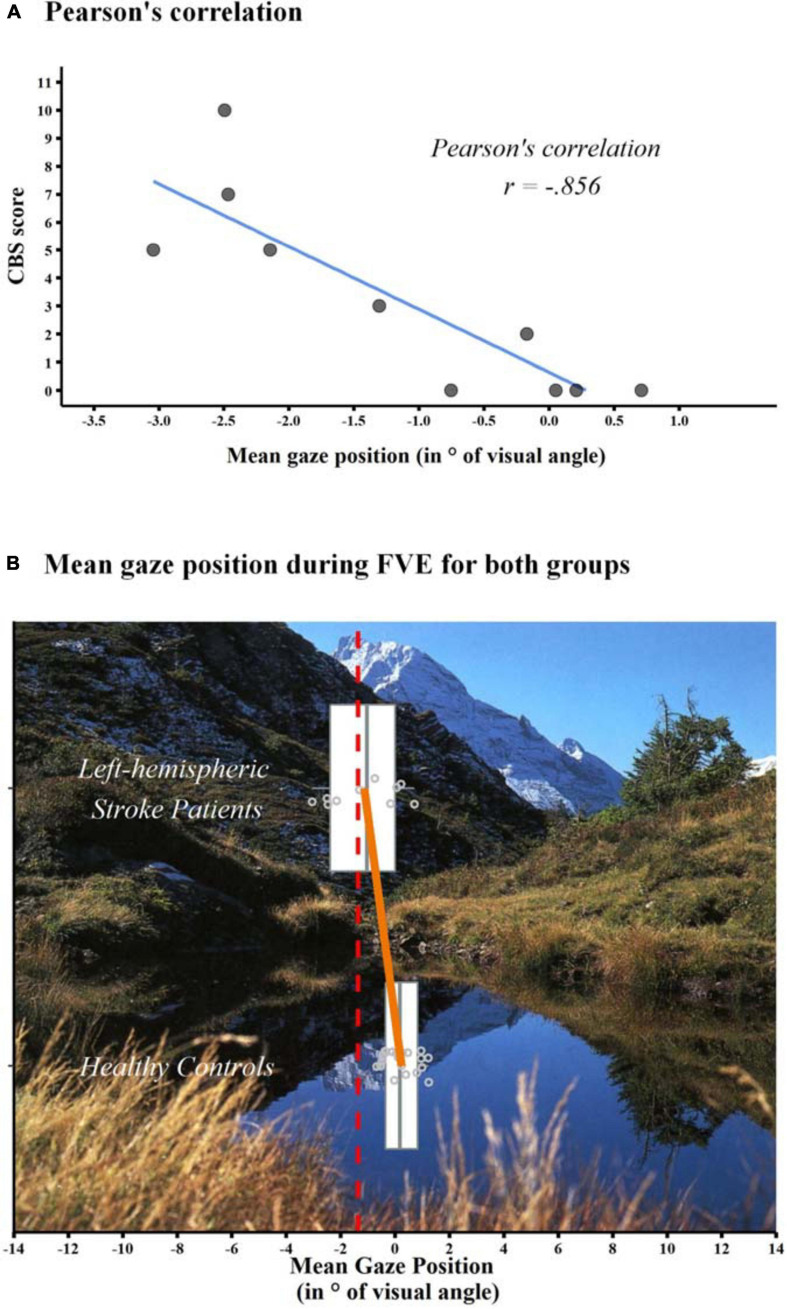
**(A)** Depiction of the Pearson’s correlation between the mean gaze position during FVE and the CBS total scores in left-hemispheric stroke patients. The regression line is shown in blue. Grey dots represent the patients’ individual CBS total scores (*y*-axis) and the corresponding mean gaze position during FVE in ° of visual angle (*x*-axis; negative values representing a left-ward shift in exploration behaviour). **(B)** Box-and-Whisker plots of the Mean Horizontal Gaze Position (in ° of visual angle) in left-hemispheric stroke patients (*n* = 10, top) and healthy controls (*n* = 20, bottom). Each box represents the lower (Q1) to the upper (Q3) quartiles, with whiskers extending to the minimum and maximum of 1.5 times the interquartile range; the grey vertical lines represent the median. Additionally, mean values of the two groups are indicated by the endpoints of the orange line, and individual values by grey dots. The red, dashed line indicates the cut-off for neglect, i.e., <–1.359° (Kaufmann et al., 2020a). An exemplary picture, used in the FVE paradigm, is shown in the background.

Pearson’s correlations in left-hemispheric stroke patients showed a significant, strong relationship between mean gaze position during FVE and CBS scores (*r* = −0.856, *p* = 0.002, Bonferroni-corrected; Figure 3A). Overall, patients with more pronounced leftward shift in FVE (i.e., lower values in the FVE) showed more severe neglect signs during everyday behaviour (i.e., higher CBS scores). Pearson’s correlations between the CBS score and the linguistic assessments did not reveal significant (Token Test; *r* = −0.140, *p* = 0.700; Boston Naming Test; *r* = −0.153. *p* = 0.672). Also, Pearson’s correlations between mean gaze position during FVE and the linguistic assessments did not show any significant results (Token Test; *r* = 0.092, *p* = 0.800; Boston Naming Test; *r* = 0.207. *p* = 0.566).

Comparing the mean gaze position (in ° of visual angle) between groups, a significant leftward shift, indicating a neglect toward the right, was found in left-hemispheric stroke patients (*t*-test for independent groups: *t*(10.994) = 3.089, *p* = 0.010; left-hemispheric stroke patients mean = −1.141, *SD* = 1.334; healthy control participants mean = 0.231, *SD* = 0.621, Figure 3B).

Comparing the percentage of left-ward first fixations between groups, a significant leftward shift, indicating a neglect toward the right, was found in left-hemispheric stroke patients (*t*-test for independent groups: *t*(28) = 3.927, *p* = 0.001; left-hemispheric stroke patients mean = 78.338%, *SD* = 18.510%; healthy control participants mean = 54.579%, *SD* = 14.040%).

**FIGURE 4:**
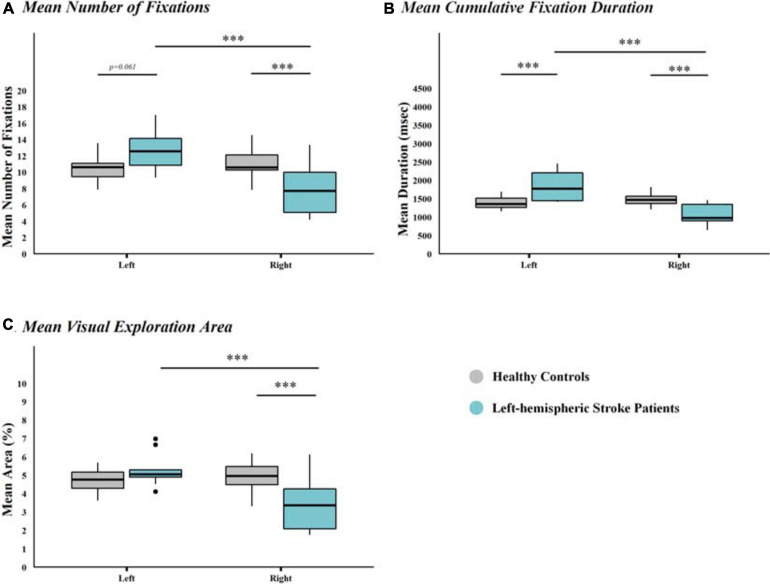
Mean Number of Fixations, Mean Cumulative Fixation Duration, and Mean Visual Exploration Area, per Group and Screen half. Box-and-Whisker plots of **(A)** mean number of fixations, **(B)** mean cumulative fixation duration, and **(C)** mean visual exploration area, per Group and Screen half. Results of left-hemispheric stroke patients (*n* = 10) are depicted in light blue, those of healthy controls (*n* = 20) in grey. Significant *post hoc* differences, as evidence by Bonferroni-corrected *t*-test, between the different combinations of factor levels are depicted by asterisks (^∗∗∗^*p* < 0.001). Median values per group are indicated by the horizontal black line in each box. Each box represents the lower (Q1) to the upper (Q3) quartiles, with whiskers extending to the minimum and maximum of 1.5 times the interquartile range. Outliers are indicated by black dots.

In left-hemispheric stroke patients, a leftward shift was also found when analysing the mean number of fixations per screen half (mixed-model ANOVA with a significant interaction between factors Group ^∗^ Screen Half; *F*_(28, 1)_ = 19.398, *p* < 0.001;η^2^ = 0.271, i.e., large effect; Figure 4A; *for a more fine-grained scale of the mean number of fixation please see* Supplementary Figure 1), the mean cumulative fixation duration (mixed-model ANOVA with a significant interaction between factors Group ^∗^ Screen Half; *F*_(28, 1)_ = 24.674, *p* < 0.00;η^2^ = 0.435, i.e., large effect; Figure 4B), and the mean visual exploration area (mixed-model ANOVA with a significant interaction between factors Group ^∗^ Screen Half [*F*_(28, 1)_ = 23.440, *p* < 0.001; η^2^ = 0.2261, i.e., large effect, Figure 4C).

## Discussion

In the present study, we show that video-oculography during a FVE paradigm is a feasible approach to evaluate right-sided neglect in left-hemispheric stroke patients with aphasia. We show that, by combining verbal and non-verbal instructions (i.e., including a step-by-step demonstration on how the measurement is conducted), high quality oculomotor data can be collected even in patients with severe aphasia.

The calibration of the eye-tracking system and its validation could be easily performed and showed reliable data quality, i.e., there was no difference between healthy participants and left-hemispheric stroke patients in this respect. Furthermore, the number of fixations excluded during offline data pre-processing was comparable to the one of studies including right-hemispheric stroke patients (e.g., in the present study 4.89%, in Kaufmann et al., 2020a about 6.25%, in Kaufmann et al., 2020b about 7.89% of all fixations were excluded).

In contrast to conventional neuropsychological neglect screenings, which depend on structured and sometimes complex verbal task instructions (i.e., Cancellation tests), virtually no explicit instructions are needed to perform FVE. Indeed, FVE is spontaneous and largely relies on automatic, bottom-up orienting of attention, similarly to every day behaviour (Pflugshaupt et al., 2004; Paladini et al., 2019). The generally positive feedback of our left-hemispheric stroke patients with aphasia and their relatives showed that the FVE paradigm and the eye movement measurement was well tolerated, and the patients felt comfortable during the assessment.

Video-oculography during FVE revealed a significant leftward shift in mean gaze position, which was highly correlated with neglect severity in everyday behaviour, as assessed by means of the CBS. Furthermore, we observed exactly the mirrored pattern that is usually observed in patients with left-sided neglect after right-hemispheric stroke, i.e., a rightward shift in exploration behaviour (e.g., Delazer et al., 2018; Kaufmann et al., 2020a).

Further indicators of lateral biases in the spatial allocation of visual attention (i.e., percentage of left-ward first fixations, mean number of fixations, mean cumulative fixation duration, and mean visual exploration area) also revealed a typical neglect pattern: early orientation toward the left and significantly fewer fixations, less time spent exploring, and a significantly smaller visual exploration area within the right half of the screen.

Finally, as an observation, it is interesting to note that video-oculography during FVE correctly identified more neglect patients after left-hemispheric stroke (4 patients) than the Bells Cancellation Test (1 patient). The present study only aimed to test the feasibility of video-oculography in aphasic patients. As a next step it might therefore be interesting to investigate the sensitivity of FVE in detecting neglect after left-hemispheric stroke in a larger patient sample similarly as it has been done in right-hemispheric stroke patients (Kaufmann et al., 2020a).

## Conclusion

In conclusion, our study shows that video-oculography is a feasible approach to diagnose right-sided neglect in left-hemispheric stroke patients, in which the compliance with verbal test instructions is often compromised by language deficits, such as aphasia. Furthermore, video-oculography during FVE may have the potential to be applied as a complementary tool for the longitudinal assessments of neglect severity in aphasic patients, as well as an outcome measure in visual attention research in left-hemispheric stroke.

## Data Availability Statement

In order to conform to the data privacy statements signed by our participants, raw individual participant data collected in this study cannot be distributed openly. However, specific aspects of the anonymized, aggregated datasets supporting the results presented in this manuscript will be shared upon request to the corresponding author.

## Ethics Statement

All patients signed a written informed general consent allowing the retrospective use of their anonymized health-related data for research purposes. Additional ethical review and approval were not required for the study on human participants in accordance with the local legislation and institutional requirements. Additionally, video-oculography data of 20 healthy, age-matched control participants (described in Data Pre-processing and Statistical Analyses), was used to investigate the feasibility of video-oculography. The data of healthy control participants has been previously included in a methodological paper, which was reviewed and approved by the Ethics Committee Nordwest and Zentralschweiz (EKNZ), Switzerland. Written informed consent to participate in this study was provided by all healthy participants.

## Author Contributions

BK, TNy, and MK-B contributed to conception and design of the study. BK and MK-B performed the assessments. BK performed the statistical analyses. BK, DC, and RM wrote the first draft of the manuscript. TNy, TNf, and MK-B wrote sections of the manuscript. DC, TNy, TNf, RM, and MK-B critically revised the work for important intellectual content. All authors contributed to manuscript revision, and read and approved the submitted version.

## Conflict of Interest

The authors declare that the research was conducted in the absence of any commercial or financial relationships that could be construed as a potential conflict of interest.

## Supplementary Material

Supplementary Figure 1 The histogram shows the spatial distribution of fixations during FVE over horizontal bins of 2° visual angle. The mean number of fixations per bin shows a left-ward shift in exploration behaviour for the left-hemispheric stroke patients (blue) compared to healthy controls (grey).

## References

[B1] Azouvi P. Olivier S. de Montety G. Samuel C. Louis-Dreyfus A. Tesio L. (2003). Behavioral assessment of unilateral neglect: study of the psychometric properties of the catherine bergego scale. *Arch. Phys. Med. Rehabil.* 84 51–57. 10.1053/apmr.2003.50062 12589620

[B2] Azouvi P. Samuel C. Louis-Dreyfus A. Bernati T. Bartolomeo P. Beis J. M. (2002). Sensitivity of clinical and behavioural tests of spatial neglect after right hemisphere stroke. *J. Neurol. Neurosurg. Psychiatry* 73 160–166. 10.1136/jnnp.73.2.160 12122175 PMC1737990

[B3] Beis J. Keller C. Morin N. Bartolomeo P. Bernati T. Chokron S. (2004). Right spatial neglect after left hemisphere stroke: qualitative and quantitative study. *Neurology* 63 1600–1605. 10.1212/01.wnl.0000142967.60579.32 15534242

[B4] Beume L. Martin M. Kaller C. Klöppel S. Schmidt C. Urbach H. (2017). Visual neglect after lefthemispheric lesions: a voxelbased lesion–symptom mapping study in 121 acute stroke patients. *Exp. Brain Res.* 235 83–95. 10.1007/s00221-016-4771-9 27637595

[B5] Bowen A. McKenna K. Tallis R. (1999). Reasons for variability in the reported rate of occurrence of unilateral spatial neglect after stroke. *Stroke* 30 1196–1202. 10.1161/01.str.30.6.1196 10356099

[B6] Carpenter R. H. (1988). *Movements of the Eyes*, 2nd Edn. London: Pion Limited.

[B7] Cazzoli D. Nyffeler T. Hess C. W. Müri R. M. (2011). Vertical bias in neglect: a question of time? *Neuropsychologia* 49 2369–2374. 10.1016/j.neuropsychologia.2011.04.010 21530558

[B8] Chung S. Park E. Ye B. Lee H. Chang H. Song D. (2016). The computerized table setting test for detecting unilateral neglect. *PLoS One* 11:e0147030. 10.1371/journalpone.0147030 26771512 PMC4714760

[B9] Delazer M. Sojer M. Ellmerer P. Boehme B. Benke T. (2018). Eye-Tracking provides a sensitive measure of exploration deficits after acute right MCA stroke. *Front. Neurol.* 9:359. 10.3389/fneur.2018.00359 29942277 PMC6004522

[B10] Fellrath J. Ptak R. (2015). The role of visual saliency for the allocation of attention: evidence from spatial neglect and hemianopia. *Neuropsychologia* 73 70–81. 10.1016/j.neuropsychologia.2015.05.003 25956677

[B11] Gauthier L. Dehaut F. Joanette Y. (1989). The bells test: a quantitative and qualitative test for visual neglect. *Int. J. Clin. Neuropsychol.* 11 49–54.

[B12] Huber W. Poeck K. Willmes K. Weniger D. (1983). *Aachener Aphasie Test (AAT).* Göttingen: Hogrefe.

[B13] Kaufmann B. Cazzoli D. Pflugshaupt T. Bohlhalter S. Vanbellingen T. Müri R. (2020a). Eyetracking during free visual exploration detects neglect more reliably than paper-pencil tests. *Cortex* 129 223–235. 10.1016/j.cortex.2020.04.021 32512414

[B14] Kaufmann B. Knobel S. Nef T. Müri R. Cazzoli D. Nyffeler T. (2020b). Visual exploration area in neglect: a new analysis method for video-oculography data based on foveal vision. *Front. Neurosci.* 13:1412. 10.3389/fnins.2019.01412 32038129 PMC6987148

[B15] Lang C. Dehm A. Dehm B. Leuschner T. (1999). *Kurze Aphasieprüfung KAP.* Frankfurt: Swets & Zeitlinger.

[B16] Morris J. Mohs R. Rogers H. Fillenbaum G. Heyman A. (1988). Consortium to Establish a Registry for Alzheimer’s Disease (CERAD) clinical and neuropsychological assessment of Alzheimer’s Disease. *Psychopharmacol. Bull.* 24 641–652. 3249766

[B17] Müri R. M. Cazzoli D. Nef T. Mosimann U. P. Hopfner S. Nyffeler T. (2013). Non-invasive brain stimulation in neglect rehabilitation: an update. *Front. Hum. Neurosci.* 7:248. 10.3389/fnhum.2013.00248 23772209 PMC3677145

[B18] Nyffeler T. Cazzoli D. Wurtz P. Lüthi M. Von Wartburg R. Vhaves S. (2008). Neglect- like visual exploration behaviour after theta burst transcranial magnetic stimulation of the right posterior parietal cortex. *Eur. J. Neurosci.* 27 1809–1813. 10.1111/j.1460-9568.2008.06154.x 18371083

[B19] Osandón J. P. Onat S. Cazzoli D. Nyffeler T. Müri R. König P. (2012). Unmasking the contribution of low-level features to the guidance of attention. *Neuropsychologia* 50 3478–3487. 10.1016/j.neuropsychologia.2012.09.043 23044277

[B20] Paladini R. Wyss P. Kaufmann B. Urwyler P. Nef T. Cazzoli D. (2019). Re-fixation and perseveration patterns in neglect patients during free visual exploration. *Eur. J. Neurosci.* 49 1244–1252. 10.1111/ejn.14309 30561071

[B21] Pflugshaupt T. Bopp S. A. Heinemann D. Mosimann U. P. von Wartburg R. Nyffeler T. (2004). Residual oculomotor and exploratory deficits in patients with recovered hemineglect. *Neuropsychologia* 42 1203–1211. 10.1016/j.neuropsychologia.2004.02.002 15178172

[B22] Ptak R. Golay L. Müri R. M. Schnider A. (2009). Looking left with left neglect: the role of spatial attention when active vision selects local image features for fixation. *Cortex* 45 1156–1166. 10.1016/j.cortex.2008.10.001 19038381

[B23] Ringman J. M. Saver J. L. Woolson R. F. Clarke W. R. Adams H. P. (2004). Frequency, risk factors, anatomy, and course of unilateral neglect in an acute stroke cohort. *Neurology* 63 468–474. 10.1212/01.WNL.0000133011.10689.CE 15304577

[B24] Rorden C. Karnath H. O. (2010). A simple measure of neglect severity. *Neuropsychologia* 48 2758–2763. 10.1016/j.neuropsychologia.2010.04.018 20433859 PMC3129646

[B25] Salthouse T. A. Ellis C. L. (1980). Determinants of eye-fixation duration. *Am. J. Psychol.* 93 207–234. 10.2307/1422228 7406068

[B26] Stapleton T. Ashburn A. Stack E. (2000). A pilot study of attention deficits, balance control and falls in the subacute stage following stroke. *Clin. Rehabil.* 15 437–444. 10.1191/026921501678310243 11518445

[B27] Stone S. P. Halligan P. W. Greenwood R. J. (1993). The incidence of neglect phenomena and related disorders in patients with an acute right or left hemisphere stroke. *Age Ageing* 21 48–52. 10.1093/ageing/22.1.46 8438666

[B28] Suchan J. Rorden C. Karnath H. (2012). Neglect severity after left and right brain damage. *Neuropsychologia* 50 1136–1141. 10.1016/j.neuropsychologia.2011.12.018 22230231 PMC3348265

