# Supplementary figures and images for: Video-Oculography During Free Visual Exploration to Detect Right Spatial Neglect in Left-Hemispheric Stroke Patients With Aphasia: A Feasibility Study
Source: Front Neurosci. 2021 Mar 29;15:640049. doi: 10.3389/fnins.2021.640049 (PMC8039453; doi:10.3389/fnins.2021.640049)

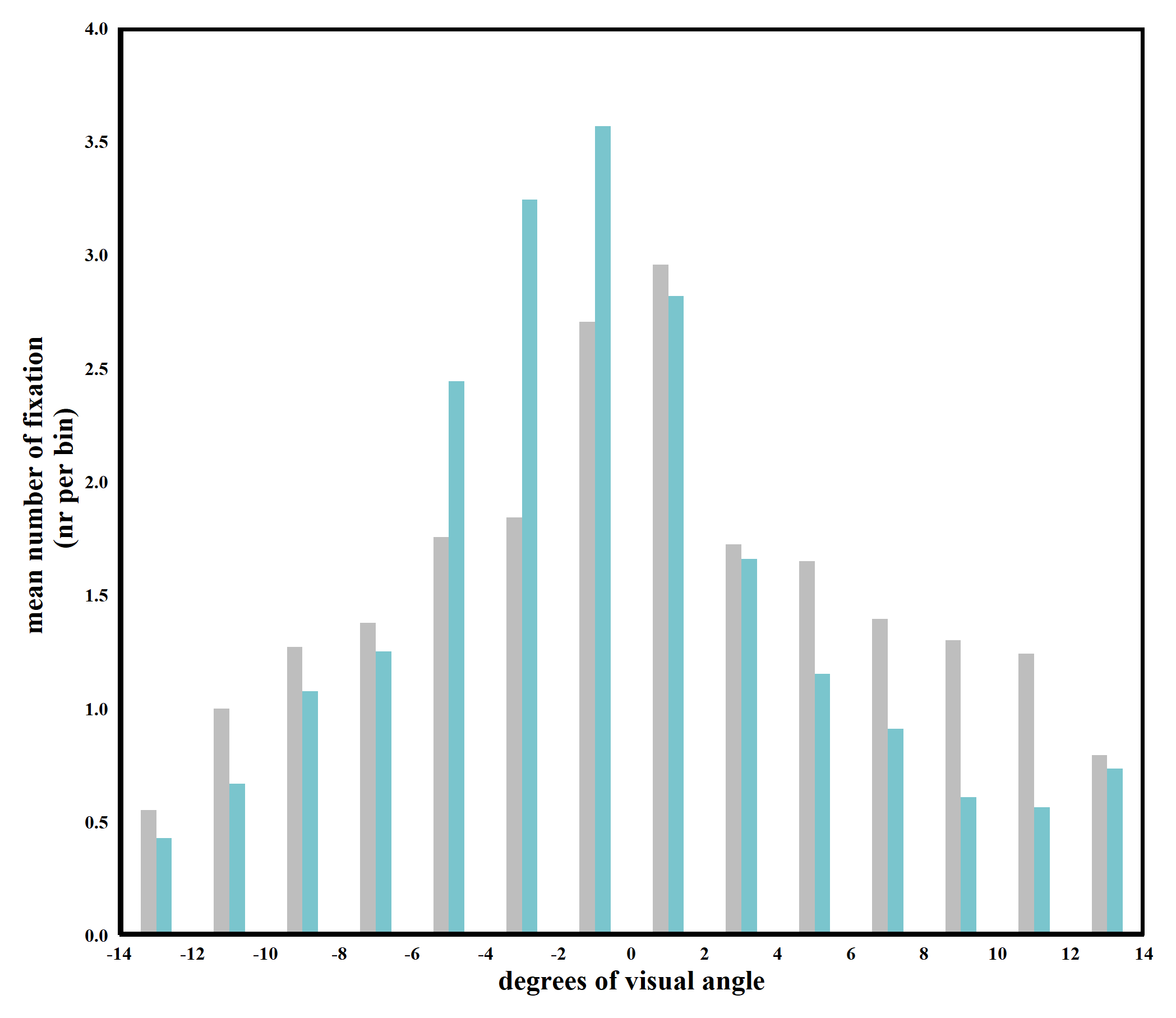

Supplement: Supplementary Figure 1 — The histogram shows the spatial distribution of fixations during FVE over horizontal bins of 2° visual angle. The mean number of fixations per bin shows a left-ward shift in exploration behaviour for the left-hemispheric stroke patients (blue) compared to healthy controls (grey). [file Image_1.TIF]
